# SIRT1 Inhibition-Induced Mitochondrial Damage Promotes GSDME-Dependent Pyroptosis in Hepatocellular Carcinoma Cells

**DOI:** 10.1007/s12033-023-00964-z

**Published:** 2023-12-03

**Authors:** Di Liu, Junhao Liu, Kejun Liu, Yanchao Hu, Jinming Feng, Yang Bu, Qi Wang

**Affiliations:** 1 Department of Hepatobiliary Surgery, General Hospital of Ningxia Medical University, 804 Shengli Street, Xingqing District, Yinchuan City, Ningxia Hui Autonomous Region China; 2 School of Clinical Medicine, Ningxia Medical University, Yinchuan, China; 3 Department of Infectious Diseases, General Hospital of Ningxia Medical University, Yinchuan, China; 4 Department of Surgery, Shapotou District People’s Hospital, Zhongwei City, China; 5 Department of Hepatobiliary Surgery, People’s Hospital of Ningxia Hui Autonomous Region, No.301, Zhengyuan North Street, Jinfeng District, Yinchuan City, Ningxia Hui Autonomous Region China

**Keywords:** Hepatocellular carcinoma (HCC), SIRT1, Mitochondrial damage, GSDME-dependent pyroptosis

## Abstract

Hepatocellular carcinoma (HCC) is a malignant tumor that affects the liver and poses a significant threat to human health. Further investigation is necessary to fully understand the role of SIRT1, a protein linked to tumorigenesis, in HCC development. To investigate the effect of SIRT1 on HCC and elucidate the underlying mechanism. Eight pairs of HCC and paracancerous normal tissue specimens were collected. The levels of SIRT1 and GSDME in tissue samples were assessed using immunohistochemistry and western blotting. SIRT1 levels were determined in HCC (Huh7, HepG2, SNU-423, SNU-398, and HCCLM3) and L-02 cells using reverse transcription-quantitative polymerase chain reaction (RT-qPCR) and western blotting. SNU-423 and HCCLM3 cells were transfected with si-SIRT1 and/or si-GSDME to knock down SIRT1 or GSDME expression. RT-qPCR and western blotting were performed to measure the expression of SIRT1, pro-casp-3, cl-casp-3, GSDME, GSDME-N, PGC-1α, Bax, and cytochrome c (Cyto C). Cell proliferation, migration, invasion, and apoptosis were assessed using the cell counting kit-8 (CCK-8), wound healing assay, Transwell invasion assay, and flow cytometry, respectively. The release of lactate dehydrogenase (LDH) was evaluated using an LDH kit. SIRT1 was upregulated in HCC tissues and cells, and a negative correlation was observed between SIRT1 and GSDME-N. SIRT1 silencing suppressed the proliferation, migration, and invasion of HCC cells while also promoting apoptosis and inducing mitochondrial damage. Additionally, the silencing of SIRT1 resulted in the formation of large bubbles on the plasma membrane of HCC cells, leading to cellular swelling and aggravated GSDME-dependent pyroptosis, resulting in an increase in LDH release. Inhibition of GSDME reduced SIRT1 silencing-induced cell swelling, decreased LDH release rate, and promoted apoptosis. SIRT1 silencing promotes GSDME-dependent pyroptosis in HCC cells by damaging mitochondria.

## Introduction

Hepatocellular carcinoma (HCC) is a prevalent and highly lethal malignancy [1] that ranks fifth in incidence and third in mortality among all malignancies [2]. The treatment options for HCC include surgical resection, hepatic artery chemotherapy thrombus, liver transplantation, and radiofrequency ablation [3]. Surgical resection is the preferred treatment for HCC, but many patients miss the optimal window for surgical intervention because of late-stage diagnoses, leading to a high mortality rate [4]. Liver transplantation has achieved satisfactory results, but is greatly limited by the scarcity of donor livers [5]. Methods such as hepatic arterial chemoembolization and radiofrequency ablation have demonstrated effective curative effects in HCC treatment. However, owing to the subtle onset of this disease, only approximately 30–40% of patients are eligible for curative treatment. Consequently, most patients with advanced HCC undergo palliative care [6]. The limitations of various therapeutic approaches, such as disease recurrence and metastasis, as well as the severity of liver disease and treatment failure, continue to hinder the effectiveness of existing therapeutic strategies. Therefore, there is an urgent need to develop novel therapeutic approaches based on the pathogenesis of HCC to improve the prognosis of patients with HCC.

Sirtuin 1 (SIRT1) is a protein with a wide tissue distribution in mammals and is homologous to yeast chromatin silencing information factor regulator 2 [7]. SIRT1 is a crucial ganglion-modulating protein involved in various physiological processes, such as aging, inflammation, oxidative stress, mitochondrial function protection, metabolic regulation, and tumor formation [8]. Recent studies have indicated that SIRT1 promotes HCC onset and progression [9–11]. SIRT1 has been found to be beneficial for HCC and drug resistance [12, 13]. However, the clinical significance and mechanism of action of SIRT1 in HCC remain unclear.

Pyroptosis, a recently discovered mode of cell death, is characterized by caspase-mediated cell lysis. Pyroptosis can be categorized into caspase-1/4/5/11-mediated GSDMD-dependent pyroptosis and caspase-3-mediated GSDME-dependent pyroptosis [14]. GSDME, a member of the gasdermin protein family, activates caspase-3 upon cell stimulation by external pathogens or death signals, leading to cleavage of the GSDME-N fragment. This results in the formation of GSDME pores, which interact with phospholipids on the cell membrane, causing the retention of cell contents, including IL-1β and IL-18, until the cell swells and eventually ruptures [15, 16]. Recent studies have extensively investigated GSDME-dependent pyroptosis, mediated by caspase-3, in tumor progression, specifically in HCC [17–19]. Oxidative stress causes mitochondrial damage, resulting in the activation of caspase-3 and the subsequent cleavage of GSDME-N, indicating a connection between mitochondrial function and GSDME-related pyroptosis [20]. SIRT1 regulates PGC-1α deacetylation to protect mitochondrial function and inhibit Bax expression, thereby alleviating pyroptosis [21]. Additionally, SIRT1 inhibits caspase-3 activation by inhibiting Bax via PGC-1α [22, 23], suggesting that SIRT1 may affect the onset and progression of HCC through mitochondria-regulated GSDME-dependent pyroptosis.

This study aimed to investigate the role of SIRT1 and GSDME-related pyroptosis in HCC using various techniques, including immunohistochemistry, western blotting, and quantitative reverse transcription polymerase chain reaction, as well as cell counting kit-8, wound healing, transwell invasion, flow cytometry, and lactate dehydrogenase release assays. The findings of this study may contribute to the identification of novel therapeutic targets in HCC.

## Materials and Methods

### Collection of Clinical Samples

This study was approved by the Ethics Committee of General Hospital of Ningxia Medical University and the clinical experience of General Hospital of Ningxia Medical University was used. HCC tissues and paired non-tumor tissues (approximately 2 cm from the excision margin) were obtained from eight patients who underwent surgical resection or puncture for HCC at the General Hospital of Ningxia Medical University. The selection criteria were as follows: (1) histological or pathological examination confirming HCC, (2) an abdominal MRI scan performed one month before surgery, and (3) availability of the tissue of the lesion and clinical characteristics of the patients. The exclusion criteria were as follows: (1) more than a month between MRI and surgery, (2) low-quality magnetic resonance images, and (3) individuals with other forms of cancer. Among the eight patients, there were an equal number of male and females. The median age of the patients was 54 years (range, 28–87 years). All participants provided informed consent and did not undergo radiotherapy or chemotherapy. All the samples were rapidly frozen in liquid nitrogen immediately after collection and stored for future use.

### Immunohistochemical (IHC) Analysis

The expression and localization of SIRT1 in adjacent and HCC tissues were investigated using the avidin–biotin immune-peroxidase (ABC) technique for IHC analysis. After dewaxing, the samples were treated with 0.3% hydrogen peroxide for inactivation. After 10 min of microwave treatment, cells were incubated with a primary antibody against SIRT1 (1:500, ab189494) at 4 ℃ overnight. The cells were then incubated with horseradish peroxidase-labeled secondary antibody (1:200, Invitrogen, Carlsbad, CA, USA) at 25 ℃ for 1 h. The sections were stained with DAB, dehydrated in gradient alcohol, placed in xylenol, and wrapped in neutral resin to make them transparent. SIRT1 protein expression was examined under a light microscope (Zeiss, Oberkochen, Germany).

### Cell Culture and Transfection

HCC cell lines (Huh7, HepG2, SNU-423, SNU-398, and HCCLM3) and L-02 cells were obtained from American Type Culture Collection (ATCC, Manassas, VA, USA). The cells were cultured in RPMI-160 medium supplemented with 10% fetal bovine serum, 100 μg/ml penicillin, and 100 μg/ml streptomycin. Cultures were maintained in a 5% CO_2_ incubator at 37 ℃. To silence SIRT1 and GSDME, si-SIRT1 (si-SIRT1: 5′-GAUGAAGUUGACCUCCUCATT-3′) and si-GSDME (5′-GCGGTCCTATTTGATGATGAA-3′) were procured from Shanghai Jinna Pharmaceutical Co., Ltd. and Biogene (Shanghai, China), respectively. Cell transfection was performed using Lipofectamine 2000 (Invitrogen, USA) according to the manufacturer's instructions. After 48 h, western blot analysis was conducted to confirm transfection efficiency.

### Cell Counting Kit-8 (CCK-8) Assay

The transfected cells were cultured for 24 and 48 h. Next, 10 μL of the CCK-8 solution (Dojindo Laboratories, Kumamoto, Japan) was added and incubated for 3 h. Absorbance was measured at 450 nm using a Microplate Reader (Bio-Rad, USA). Cell viability was calculated based on the absorbance value of each well using the following formula: cell viability (OD) = OD of the experimental group—OD of the blank control group.

### Wound Healing Assay

Cells seeded in 12-well plates were fused and scratched using a 20 μL sterile gun tip to create a cell-free area approximately 4 mm wide. Following PBS washing, the cells were photographed at 0 and 24 h post-transfection to observe changes in their migration ability. The area of cells migrating to the blank area was measured using ImageJ software (NIH, Bethesda, MD, USA) to determine the wound healing rate.

### Transwell Invasion Assay

HCC cells were resuspended in serum-free RPMI-160 medium and the cell concentration was adjusted. For the invasion assay, a 1:6 dilution of Matrigel glue was prepared in serum-free RPMI-1640, and 50 μL of the diluent was coated onto the bottom of the transwell chamber. Subsequently, 8 × 10^4^ cells were seeded in the upper chamber and RPMI-16440 supplemented with 10% fetal bovine serum was added to the lower chamber. The cells were then incubated in the cell incubator for 24 h. After fixation and staining, six fields were randomly selected under a microscope (Zeiss, Oberkochen, Germany) and the number of cells penetrating the filter membrane was counted.

### Flow Cytometry Assay

Cells at the logarithmic growth stage were collected by centrifugation and the supernatant was discarded. Subsequently, annexin binding buffer, PI, and annexin were added to the cells, according to the manufacturer’s instructions (BD, New York, NJ, USA). Apoptosis levels were determined and analyzed using flow cytometry (BD Biosciences, Franklin Lakes, NJ, USA). The experiment was repeated three times.

### Lactate Dehydrogenase (LDH) Release Assay

The CytoTox96 LDH release kit (Promega, Madison, WI, USA) was used to evaluate the LDH activity in the cell culture supernatants of each group. LDH activity was expressed as a percentage of total LDH in the cell lysate.

### RT-qPCR

RT-qPCR was used to detect SIRT1 mRNA levels. Total RNA was extracted from HCC cells using the TRIzol method, under the following standard reaction conditions: pre-denaturation at 94 ℃ for 10 min, denaturation at 94 ℃ for 1 min, annealing at 47 ℃ for 1 min, extension at 72 ℃ for 1 min, with 30 cycles, and finally extension at 72 ℃ for 10 min. The amplified products were analyzed by 1.5% agarose gel electrophoresis. GAPDH served as the internal control for SIRT1 expression. The following primer sequences were used: SIRT1, forward:5′-TCAGCTGTTGGCTGACTTCAT-3′, reverse:5′- TCCCAATGCGATGCTGACTT-3′; GAPDH, forward:5′- CAGCCCCAGAGTGTGTATCC-3′, reverse:5′-GAAGATGCGGTCACCTCACA-3′.

### Western Blot

Cells grown in logarithmic stages were seeded in six-well plates and washed with PBS after discarding the medium. The cells were lysed using RIPA, and proteins were obtained via centrifugation. The BCA protein quantification assay (Thermo Scientific, Rockford, IL, USA) was used to determine the cellular protein concentration. Protein samples (10 μg) were analyzed using sodium dodecyl sulfate–polyacrylamide gel electrophoresis (SDS-PAGE) at a constant voltage of 120 V for 60 min. The samples were blocked with 5% non-fat milk powder and incubated overnight at 4 °C with primary antibodies against SIRT1 (1:1000, ab189494), pro-casp-3 (1:500, ab32150), cl-casp-3 (1:1000, ab32042), GSDME (1:1000, ab230482), GSDME-N (1:1000, ab215191), PGC-1α (1:1000, ab188102), Bax (1:1000, ab32503), cytochrome c (Cyto C) (1:5000, ab133504), and GAPDH (1:1000, ab8245). After washing with TBST, the samples were treated with horseradish peroxidase-labeled secondary antibodies (1:5000, ab6741) and incubated at 25 °C for 1.5 h. ECL color development was visualized using an automatic chemiluminescence imaging analysis system (Tanon, Shanghai, China) and analyzed using ImageJ software (version 1.45 s, Wayne Rasband, National Institutes of Health, USA, public domain).

### Statistical Analysis

Statistical analysis was carried out using IBM SPSS 20.0 (IBM Corporation, Armonk, NY, USA) and GraphPad Prism 7 (GraphPad software, California La Jolla, USA). The measurement data were presented as mean ± standard deviation Students' t-tests were used to compare the two groups, Pearson correlation analyses were administered to correlation analysis between SIRT1 and DSDME-N. A statistically significant difference was defined as *P* < 0.05.

## Results

### SIRT1 is Upregulated and Negatively Correlated with GSDME-Dependent Pyroptosis in HCC Tissues

**Fig. 1:**
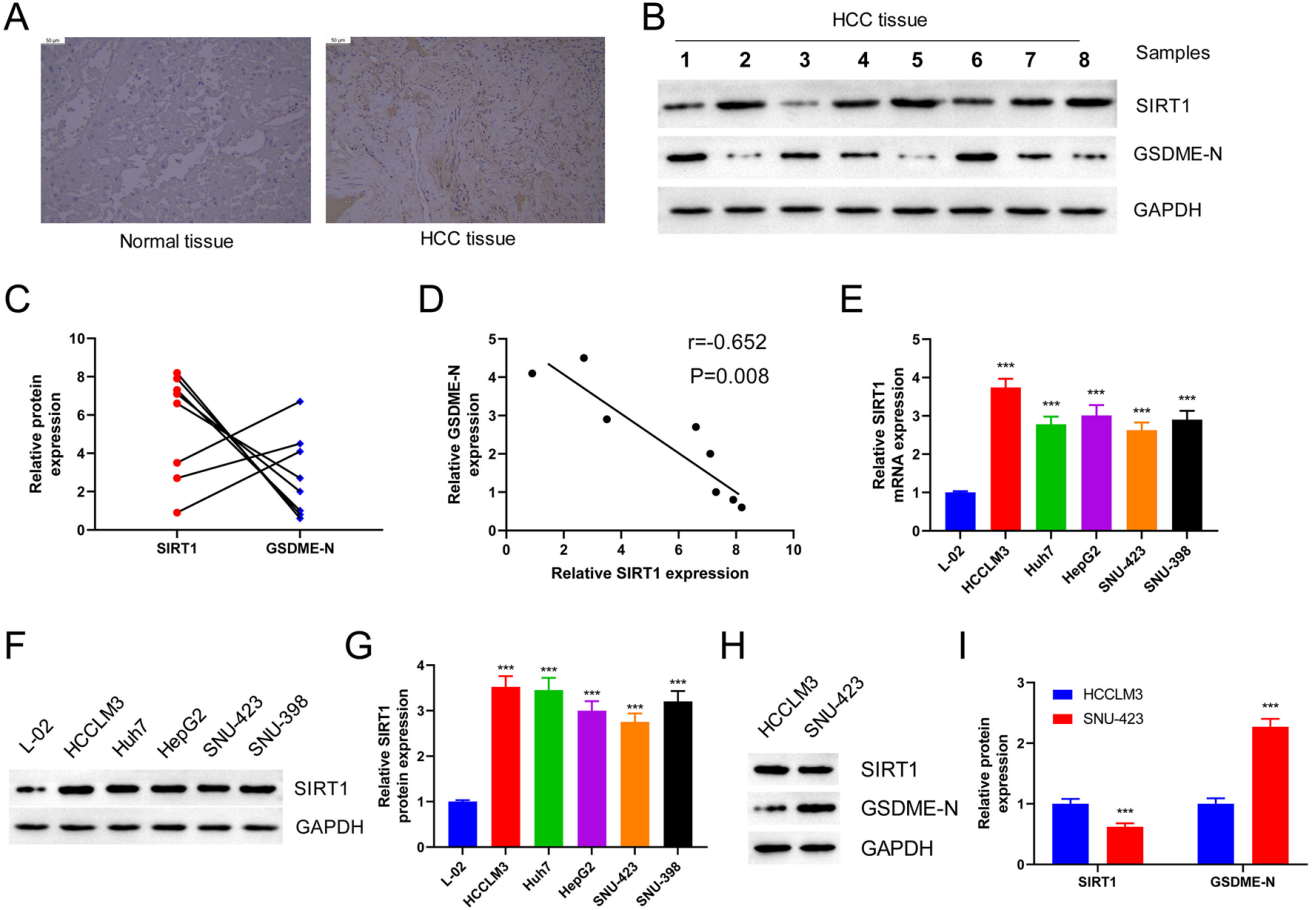
SIRT1 is upregulated and negatively correlated with GSDME-dependent pyroptosis in HCC tissues. **A** IHC was used to detect the expression and localization of SIRT1 in HCC and adjacent tissues (magnification: × 100). **B** Representative Western blot of SIRT1 protein expression in 8 HCC tissues. **C** Histogram represented the relative expression level of SIRT1 and GSDME-N protein. **D** Pearson correlation analysis was used to detect the correlation between SIRT1 and GSDME-N in HCC tissues. **E** The levels of SIRT1 in HCC cell lines (Huh7, HepG2, SNU-423, SNU-398, HCCLM3) were detected by RT-qPCR. **F** The levels of SIRT1 were detected using Western blot in Huh7, HepG2, SNU-423, SNU-398 and HCCLM3 cells. **G** Histogram represented the relative expression level of SIRT1 protein. **H** GSDME-N levels in HCC cell lines SNU-423 and HCCLM3 were detected by Western blot. **I** Histogram represented the relative expression level of SIRT1 and GSDME-N protein. ***P < 0.001 vs. L-02 group

IHC staining of SIRT1 in HCC and adjacent tissues revealed that SIRT1 was highly expressed in HCC tissues compared to adjacent tissues and was primarily localized in the nucleus (Fig. 1A). Western Blot analysis of SIRT1 and GSDME-N levels in eight HCC tissue samples revealed that SIRT1 was highly expressed in samples 2, 4, 5, 7, and 8, while GSDME-N levels were low (Fig. 1B, C). Additionally, Pearson correlation analysis confirmed a significant negative association between SIRT1 and GSDME-N expression in HCC tissues (*p* < 0.01; Fig. 1D). Furthermore, RT-qPCR (Fig. 1E) and western blot (Fig. 1F, G) analyses of SIRT1 expression in HCC cell lines (Huh7, HepG2, SNU-423, SNU-398, and HCCLM3) showed that SIRT1 expression was significantly elevated in HCC cell lines (*p* < 0.001). Specifically, SNU-423 cells exhibited relatively low SIRT1 expression, whereas HCCLM3 cells exhibited relatively high SIRT1 expression. Further investigation of the levels of GSDME-N in HCCLM3 and SNU-423 cells showed that SNU-423 cells with low SIRT1 expression had high GSDME-N expression, whereas HCCLM3 cells with high SIRT1 expression had low GSDME-N expression. (Fig. 1H, I). These findings suggested that SIRT1 was abnormally overexpressed in HCC tissues and was negatively associated with GSDME-N.

### SIRT1 Silencing Inhibits the Proliferation, Migration, and Invasion of HCC Cells

**Fig. 2:**
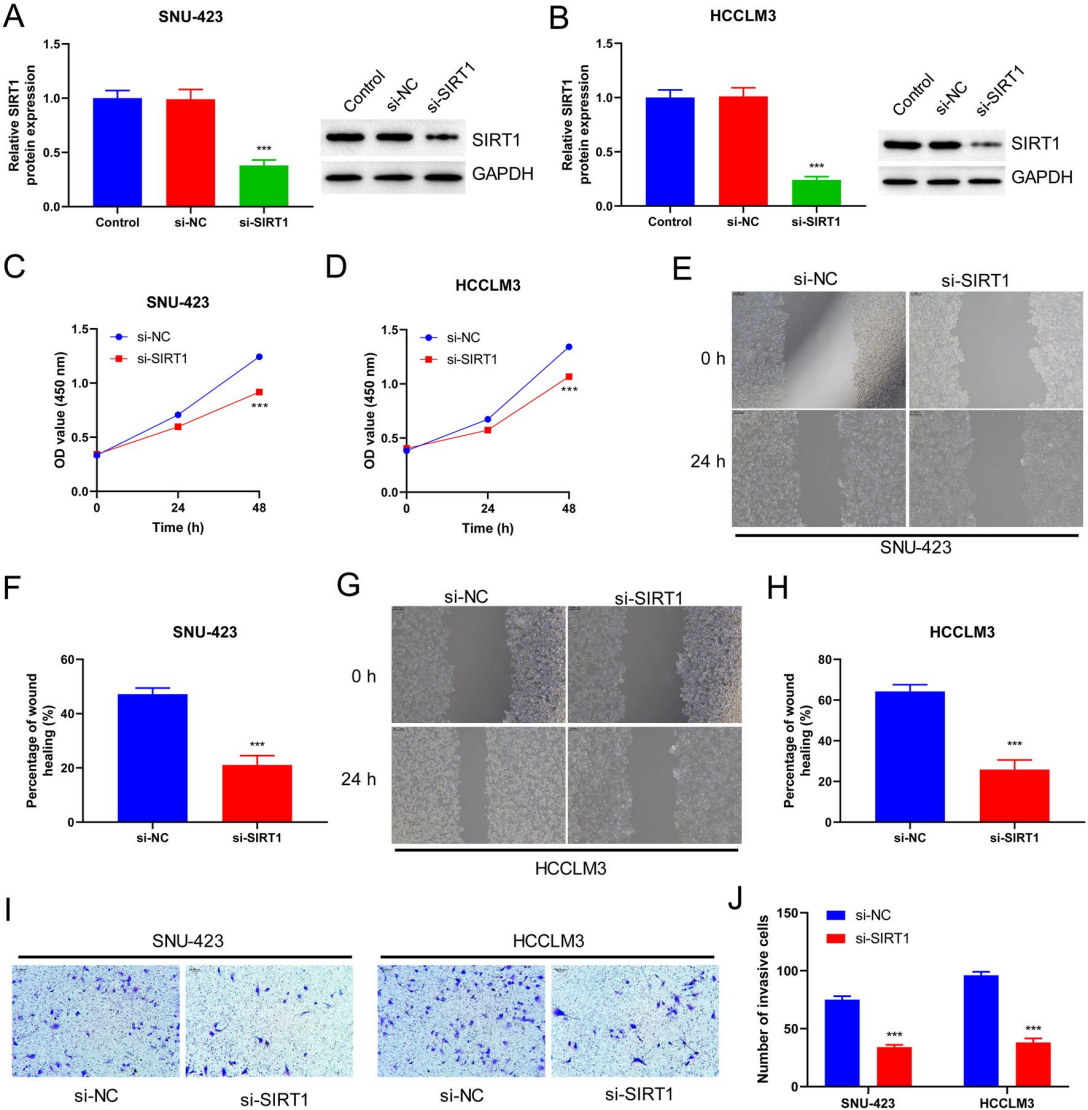
Silencing SIRT1 inhibits the proliferation, migration and invasion of HCC cells. **A** Western Blot was used to detect the protein levels of SIRT1 in SNU-423 cells after transfection with siRNA. **B** SIRT1 mRNA levels were verified by RT-qPCR. **C** CCK-8 was used to examine the effect of SIRT1 silencing on the proliferation of SNU-423 cells. **D** HCCLM3 activity was detected by CCK-8. **E** The migration ability of SNU 423 cells was examined by wound healing assay (magnification: × 100). **F** Histogram represented percentage of wound healing. **G** The migration ability of HCCLM3 cells was examined by wound healing assay (magnification: × 100). **H** Histogram represented percentage of wound healing. **I** Cell invasion was detected by transwell assay. **J** Histogram represented number of invasive cells (magnification: × 100). ***P < 0.001 vs. si-NC group

Next, we explored the effect of SIRT1 on the biological behavior of HCC cells. SIRT1 levels were significantly reduced in si-SIRT1 transfected SNU-423 (Fig. 2A) and HCCLM3 cells (Fig. 2B) compared to those in the si-NC group (*p* < 0.001). Therefore, the small interfering RNA used in this study effectively silenced the expression of SIRT1 in HCC cell lines, specifically SNU-423 and HCCLM3 cells. Subsequently, the viability of SNU-423 and HCCLM3 cells in the si-NC and si-SIRT1 groups was assessed at different time points (24 and 48 h after transfection). The cell viability of the si-SIRT1 group was significantly lower than that of the si-NC group (*p* < 0.001; Fig. 2C, D). Additionally, the migration ability of SNU-423 and HCCLM3 cells decreased significantly with SIRT1 knockdown (*p* < 0.001; Fig. 2E–H). The invasive ability of HCC cells was significantly reduced following SIRT1 knockdown (*p* < 0.001; Fig. 2I). These findings suggested that SIRT1 silencing significantly blocked the proliferation, migration, and invasion of HCC cells.

### SIRT1 Silencing Promotes Apoptosis and Induces Mitochondrial Damage in HCC Cells

**Fig. 3:**
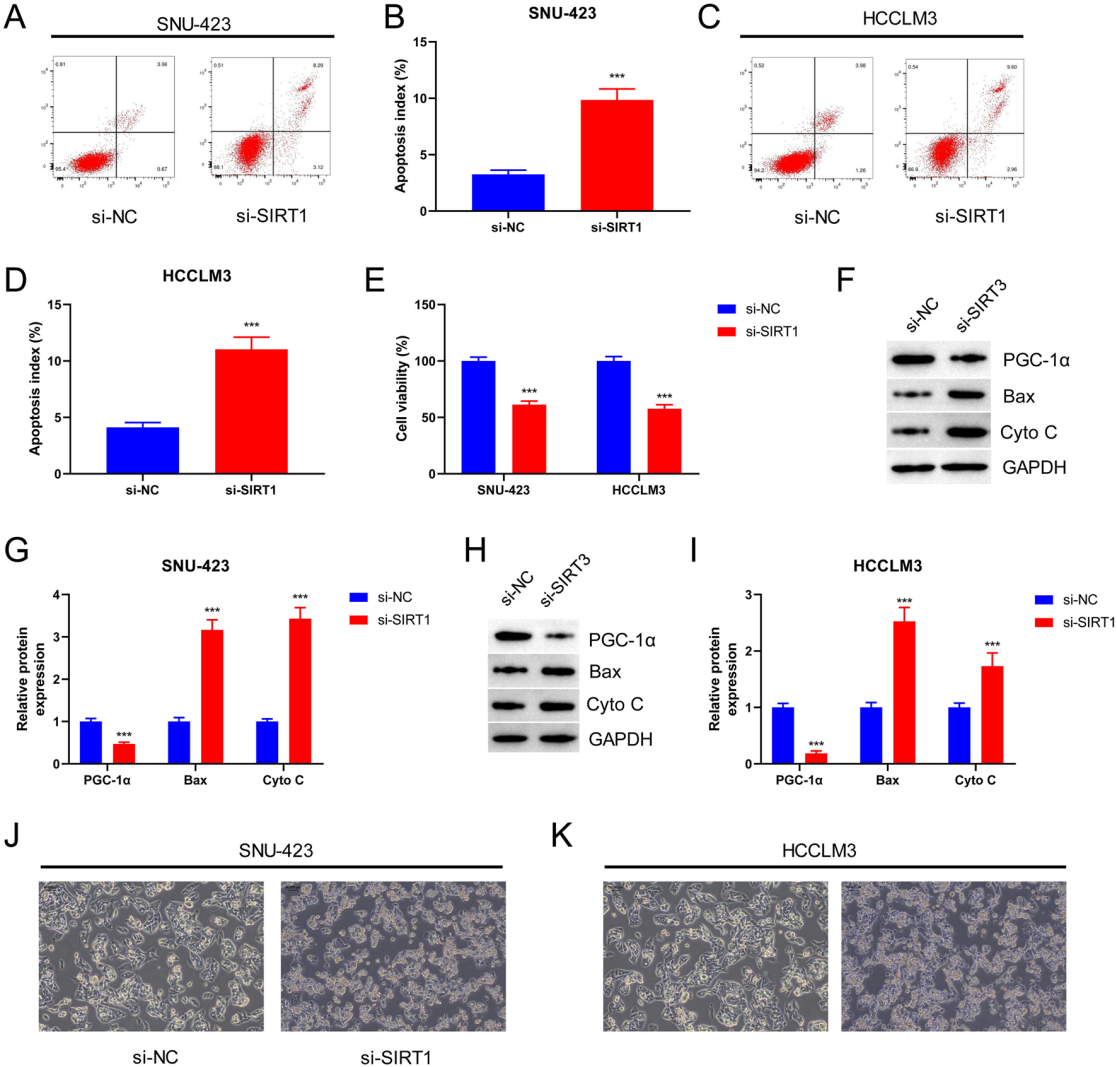
Silencing SIRT1 promotes cell apoptosis and induced mitochondrial damage of HCC cells. **A** Apoptosis of SNU-423 cells was detected by flow cytometry. **B** Histogram represented apoptosis index. **C** Apoptosis of HCCLM3 cells was verified by flow cytometry. **D** Histogram represented apoptosis index. **E** CCK-8 assay was used to measure cell viability **F** The protein levels of PGC-1α, Bax and Cyto C were detected by Western blot in SNU-423 cells. **G** Histogram represented the relative expression level of PGC-1α, Bax and Cyto C protein. **H** PGC-1α, Bax and Cyto C protein levels were detected by Western blot in HCCLM3 cell. **I** Histogram represented the relative expression level of PGC-1α, Bax and Cyto C protein. **J** Mitochondrial damage in SNU-423 cells was observed by electron microscopy (magnification: × 200). **K** Mitochondrial damage in HCCLM3 cells was observed by electron microscopy (magnification: × 200). ***P < 0.001 vs. si-NC group

Investigation of the effect of SIRT1 knockdown on apoptosis in SNU-423 and HCCLM3 cells revealed that apoptosis was significantly increased in the si-SIRT1 group, leading to a significant decrease in cell viability (*p* < 0.001; Fig. 3A–E). Moreover, western blot analysis of mitochondrial function-related proteins (PGC-1α, Bax, and Cyto C) demonstrated that PGC-1α levels were significantly reduced in the si-SIRT1 group compared to those in the si-NC group. Conversely, Bax and Cyto C levels were significantly increased (*p* < 0.001) in the si-SIRT1 group, suggesting impaired mitochondrial function in SNU-423 and HCCLM3 cells (Fig. 3E–I). Electron microscopic analysis of mitochondria revealed that both SNU-423 and HCCLM3 cells showed a significant increase in the mitochondrial damage index following interference with si-SIRT1 (Fig. 3J, K). These findings suggested that SIRT1 silencing might promote apoptosis and cause mitochondrial damage in HCC cells.

### SIRT1 Silencing Induces GSDME-Related Pyroptosis

**Fig. 4:**
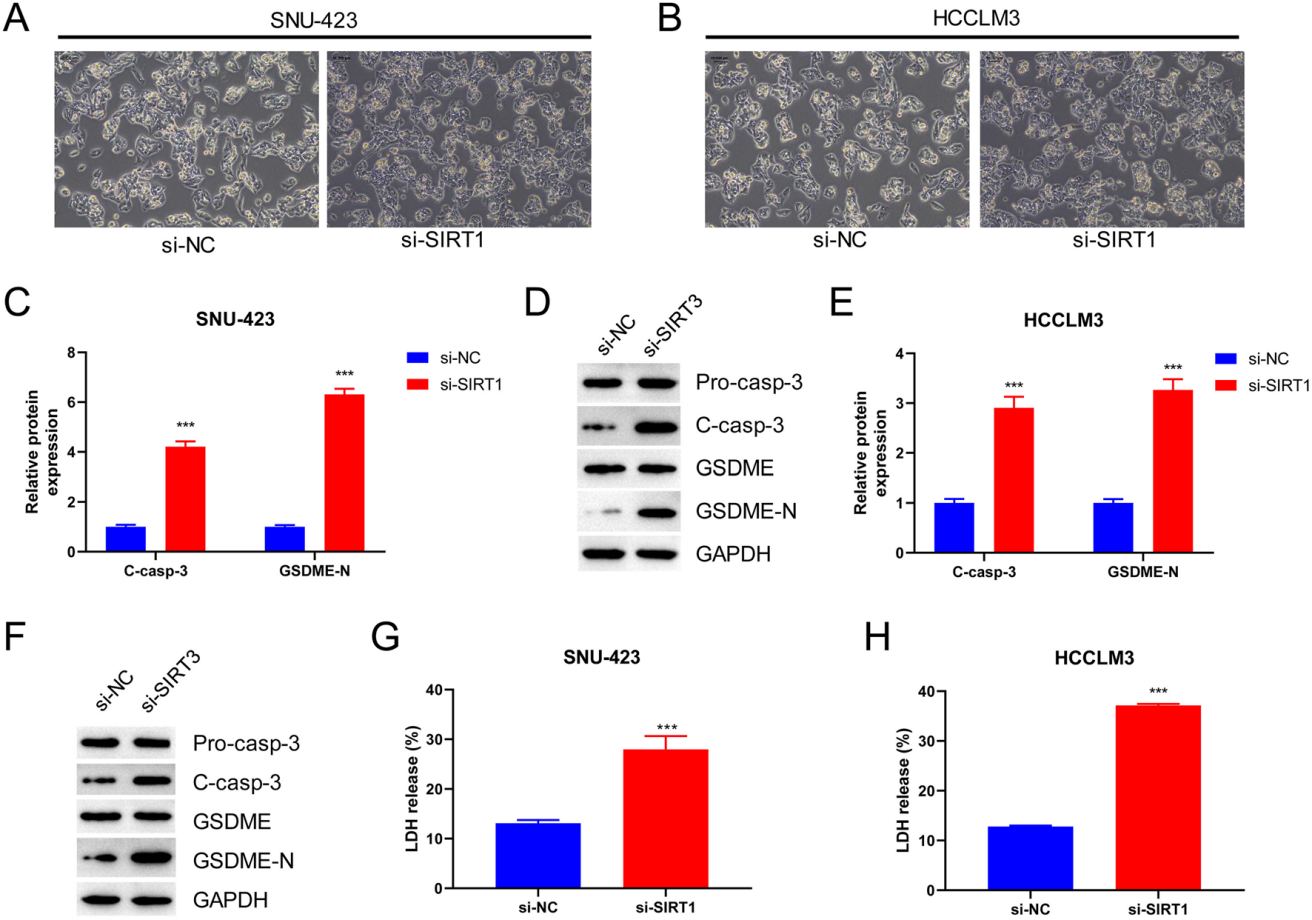
Silencing SIRT1 induces GSDME-related pyroptosis. **A** The morphology of SNU-423 cells was observed by microscope (magnification: × 200). **B** The morphology of HCCLM3 cells was observed by microscope (magnification: × 200). **C** Western blot was used to detect the level of GSDME-dependent pyroptosis markers in SNU-423 cells. **D** Histogram represented the relative expression level of C-casp-3 and GSDME-N protein. **E** The protein level of Pro-casp-3, C-casp-3, GSDME, and GSDME-N in HCCLM3 cells were detected by Western Blot. **F** Histogram represented LDH release. **G** LDH release from SNU-423 cell culture supernatant was detected with LDH assay kit. **H** LDH release from HCCLM3 cell culture supernatant was detected with LDH assay kit. ***P < 0.001 vs. si-NC group

Next, we investigated the effect of SIRT1 silencing on GSDME-related pyroptosis. Microscopic analysis of the morphology of si-SIRT1-transfected SNU-423 and HCCLM3 cells revealed swelling and numerous balloon-like protrusions (Fig. 4A, B). In addition, the expression levels of GSDME-dependent pyroptosis marker proteins, including pro-casp-3, cl-casp-3, GSDME, and GSDME-N, were significantly upregulated in the si-SIRT1 group of SNU-423 cells (*p* < 0.001; Fig. 4C, D). Similar results were observed in HCCLM3 cells (*p* < 0.001; Fig. 4E, F). Measurement of LDH release rates showed that SIRT1 silencing significantly increased LDH release in both SNU-423 and HCCLM3 cells (*p* < 0.001; Fig. 4G, H). These findings suggested that silencing SIRT1 expression could effectively induce GSDME-dependent pyroptosis.

### Inhibition of GSDME Reverses SIRT1 Silencing-Induced Pyroptosis

**Fig. 5:**
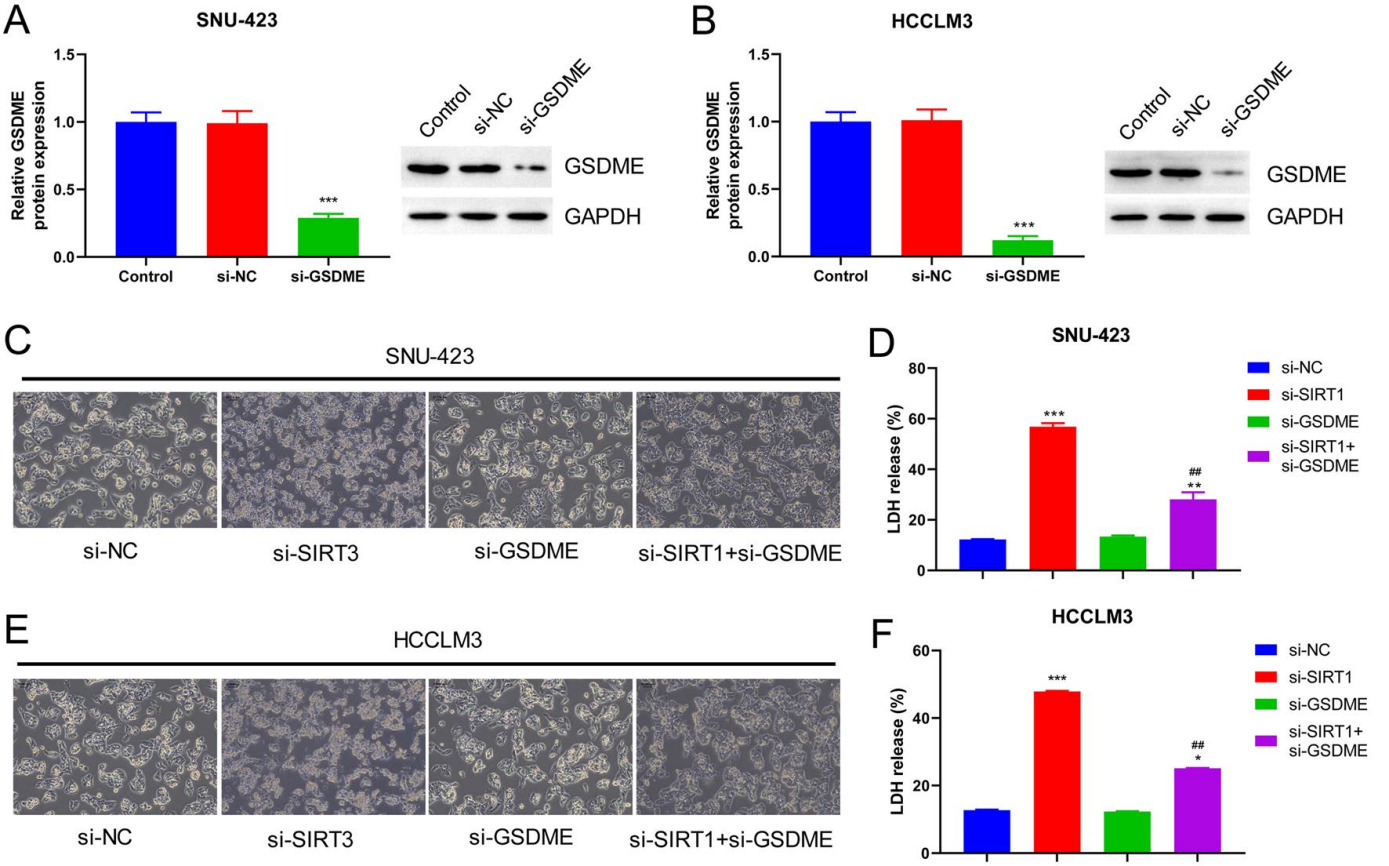
Inhibition of GSDME reverses pyroptosis induced by silencing SIRT1. **A** The protein levels of GSDME in SNU-423 cells were detected by Western Blot. **B** The protein levels of GSDME in HCCLM3 cells were detected by Western Blot. **C** The microscope was used to observe the morphology of SNU-423 cells (magnification: × 200). **D** LDH assay kit was used to detect the release of LDH in the culture supernatant of SNU-423 cells. **E** The microscope was used to observe the morphology of HCCLM3 cells (magnification: × 200). **F** LDH assay kit was used to detect the release of LDH in the culture supernatant of HCCLM3 cells. *, ***P < 0.05, 0.001 vs. si-NC group; ##*P* < 0.01 vs. si-SIRT1 group

Next, we examined the effect of GSDME on SIRT1-regulated pyroptosis. First, the transfection of GSDME was confirmed in SNU-423 and HCCLM3 cell lines (*p* < 0.001) (Fig. 5A, B). Furthermore, pyroptosis-related morphology of the cells was observed, and the LDH release rate was determined. In SNU-423 cells, the degree of cell swelling and vesicle-like protrusions were significantly reduced in the si-SIRT1 + si-GSDME group compared to that in the si-SIRT1 (Fig. 5C), and the LDH release rate was significantly reduced (*p* < 0.01, *p* < 0.001; Fig. 5D). Similar results were observed in HCCLM3 cells (*p* < 0.05, *p* < 0.01, and *p* < 0.001; Fig. 5E, F). These findings suggest that the inhibition of GSDME can effectively reverse SIRT1 knockdown-induced pyroptosis.

### Inhibition of GSDME Converts SIRT1 Silencing-Induced Pyroptosis into Apoptosis

**Fig. 6:**
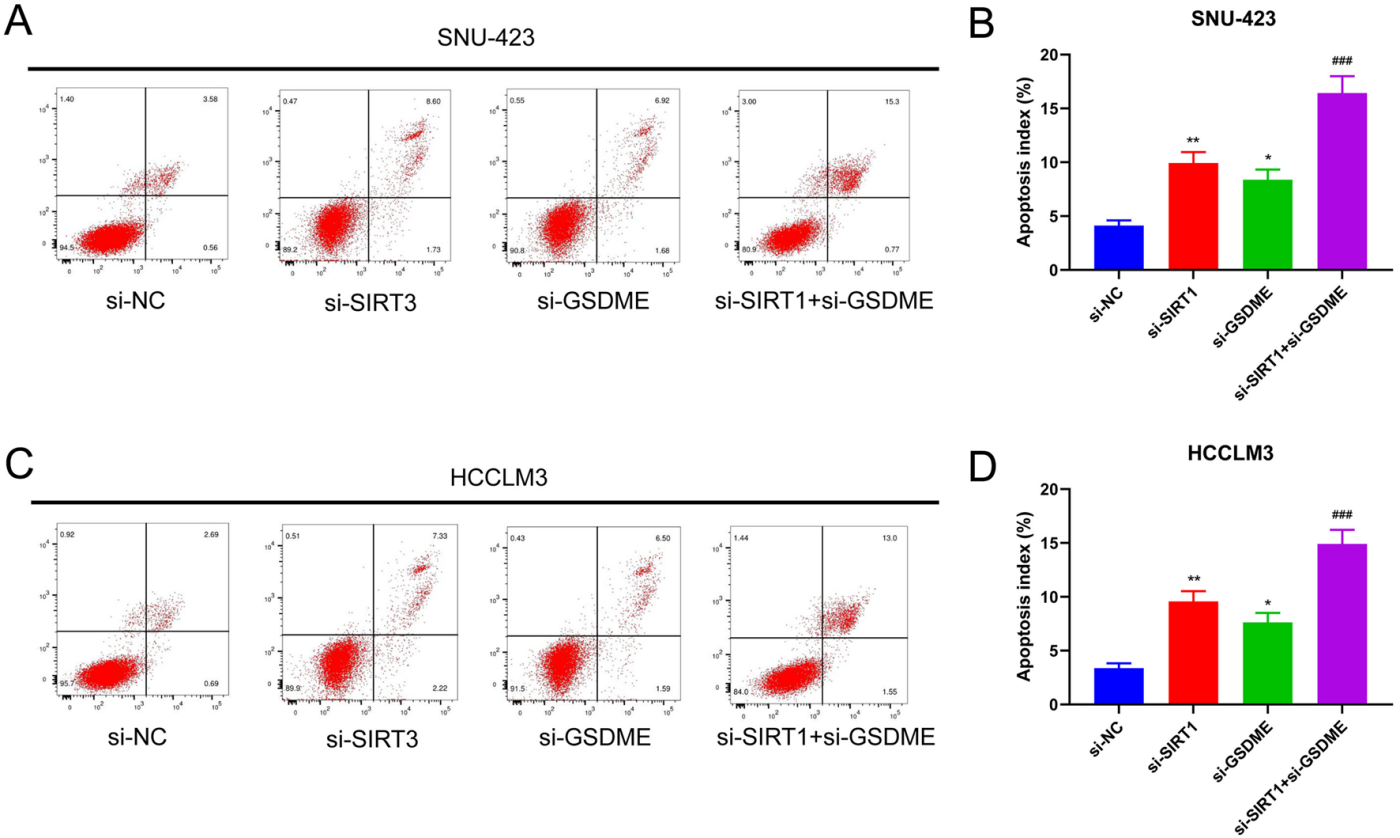
Inhibition of GSDME converts pyroptosis induced by silencing SIRT1 to apoptosis. **A** Flow cytometry was performed to detect apoptosis in different groups of SNU-423 cells. **B** Histogram represented apoptosis index. **C** Flow cytometry was used to detect apoptosis in different groups of HCCLM3 cells. **D** Histogram represented apoptosis index. *, **P < 0.05, 0.01 vs. si-NC group; ###*P* < 0.001 vs. si-SIRT1 group

Flow cytometry analysis of both SNU-423 (*p* < 0.05, *p* < 0.01, and *p* < 0.001; Fig. 6A, B) and HCCLM3 cell lines (*p* < 0.05, *p* < 0.01, and *p* < 0.001; Fig. 6C, D) revealed that apoptosis levels in the si-SIRT1 and si-GSDME groups were significantly higher than those in the control group. Furthermore, co-transfection with si-SIRT1 and si-GSDME resulted in a further increase in the depth of apoptosis. These findings suggest that the inhibition of GSDME can prevent SIRT1 knockdown-induced pyroptosis, indicating that SIRT1 deficiency leads to pyroptosis transitioning to apoptosis in HCC cells when GSDME is silenced.

## Discussion

HCC is one of the most common malignant tumors globally. HCC has a poor prognosis due to early metastasis and frequent postoperative recurrence, resulting in a 5-year postoperative survival rate of only 40–50% [24]. Further research on the molecular biology of HCC is of great significance, as novel effective treatments are necessary to improve the diagnosis and treatment of HCC patients. SIRT1 has been found to have a dual role in tumor progression, and high SIRT1 expression may promote prostate cancer [25], pancreatic cancer [26], melanoma cell invasion, and metastasis [27]. However, in oral squamous cell carcinoma, SIRT1 acts as a potential tumor suppressor [28]. This study unveiled the role of SIRT1 in HCC. Analysis of the levels of SIRT1 in HCC tissues and cells showed that SIRT1 is highly expressed in HCC tissues, consistent with the findings of Song et al. [10]. Variations in SIRT1 levels were observed in different HCC tissues, which may be attributed to two factors. First, HCC is a heterogeneous disease with variable patient characteristics. This leads to differences in screening results owing to variations in specimen collection criteria, specimen types, and patient-specific factors. Second, variations in the research methods, experimental techniques, reagents, instruments, and other interfering factors can lead to different outcomes. In addition, immunohistochemistry confirmed the strong nuclear localization of SIRT1, suggesting its role in the regulation of nuclear transcription factors through deacetylation.

The dual role of SIRT1 in tumor progression is evident from its effects on tumor cell proliferation, migration, and invasion. A previous study verified that SIRT1 overexpression promotes the proliferation of endometrial cancer cells in vitro and tumor formation in nude mice, and increases the resistance of endometrial cancer cells to cisplatin [29]. In glioma cells, miR-138 decreases SIRT1 levels by targeting the 3' non-coding region, further inhibiting cell proliferation, migration, and invasion [30]. These studies suggest that SIRT1 positively regulates tumor cell growth and metastasis. However, studies of gastric cancer have revealed that miR-132-3p promotes gastric cancer cell proliferation by targeting the 3'-UTR of SIRT1 to inhibit its expression [31]. These results indicate that SIRT1 negatively regulates tumor proliferation. In our study, SIRT1 silencing significantly inhibited the proliferation, migration, and invasion of HCC cells, while promoting cell apoptosis. These findings suggest that SIRT1 silencing inhibits HCC development.

Recently, the influence of tumor cell energy metabolism on tumor formation and progression has gained significant attention, with mitochondria playing a central role [32]. SIRT1, an NAD^+^-dependent enzyme, is closely associated with mitochondrial function and metabolism in cells and organisms, making it a distinctive sensor of cellular metabolic state [33]. Moreover, SIRT1 is increasingly being recognized as a key regulator of mitochondrial biogenesis [34]. SIRT1 activates receptor-γ coactivators via acetylated peroxisome proliferators during transcription 1α (PGC-1α) [35], which regulates the expression of downstream mitochondria-related proteins and promotes mitochondrial biosynthesis [36]. SIRT1/PGC-1α signaling participates in mitochondrial biosynthesis and metabolism [35]. PGC-lα and SIRT1 are nuclear proteins that regulate the transcription of nuclear-encoded mitochondrial genes. They are distributed in the mitochondria, exist as free proteins, and can interact with mitochondrial transcription factor A (TFAM) [37]. Mitochondria can form multiprotein complexes involving SIRT1 and PGC-lα, such as PGC-lα/SIRT1, TFAM/PGC-lα, and TFAM/SIRT1 complexes. In addition, PGC-lα and SIRT1 participate in the formation of large multiprotein complexes with TFAM. Moreover, PGC-lα positively affects TFAM activity in the D-loop region of mitochondrial DNA. Therefore, SIRT1 and PGC-1 may contribute directly or indirectly to mitochondrial biogenesis [35]. SIRT1 inhibition effectively decreased the levels of mitochondrial function-related PGC-1α, Bax, and Cyto C, leading to impaired mitochondrial function. These findings suggest that SIRT1 silencing may regulate mitochondrial damage through PGC-1α.

Caspase-3 activation triggers both apoptosis and GSDME-dependent pyroptosis. Pyroptosis is a distinct form of programmed cell death that differs from apoptosis in terms of cell membrane rupture, macrophage involvement, and inflammation. Caspase-3 activation triggers GSDME-dependent pyroptosis at high GSDME expression levels and cell apoptosis at low GSDME expression levels [38]. Additionally, GSDME-dependent pyroptosis can be triggered by intrinsic mitochondrial apoptosis [39] and is a downstream event of the mitochondria-mediated caspase pathway, which is a non-apoptotic mechanism for eliminating cancer cells [40]. In addition, Zhang et al. found that mitochondrial dysfunction can activate the caspase-3-GSDME pathway, resulting in pyroptosis of HCC cells [41]. This study demonstrated that SIRT1 silencing leads to mitochondrial damage and is negatively correlated with GSDME, which could induce GSDME-related pyroptosis. Inhibition of GSDME results in a shift from pyroptosis to apoptosis upon SIRT1 silencing. These findings suggest that silencing SIRT1 promotes the transformation of apoptosis to GSDME-dependent pyroptosis by regulating mitochondrial damage.

## Conclusion

SIRT1 silencing exhibits anti-cancer effects in the initiation and progression of HCC. The activation of GSDME-dependent pyroptosis could be a promising therapeutic approach for HCC patients with low SIRT1 levels. This study provides a novel strategy for treating HCC.

## Data Availability

The datasets generated during and/or analysed during the current study are available from the corresponding author on reasonable request.
